# Supplementary material for: A Humanized Yeast Phenomic Model of Deoxycytidine Kinase to Predict Genetic Buffering of Nucleoside Analog Cytotoxicity
Source: Genes (Basel). 2019 Sep 30;10(10):770. doi: 10.3390/genes10100770 (PMC6826991; doi:10.3390/genes10100770)
Supplement: Supplementary file 1 [file genes-10-00770-s001.zip › Additional_Files/Additional_File7_GOTermHeatmaps.zip!/Additional_File7_GOTermHeatmaps/ribosomal_small_subunit_biogenesis.pdf]

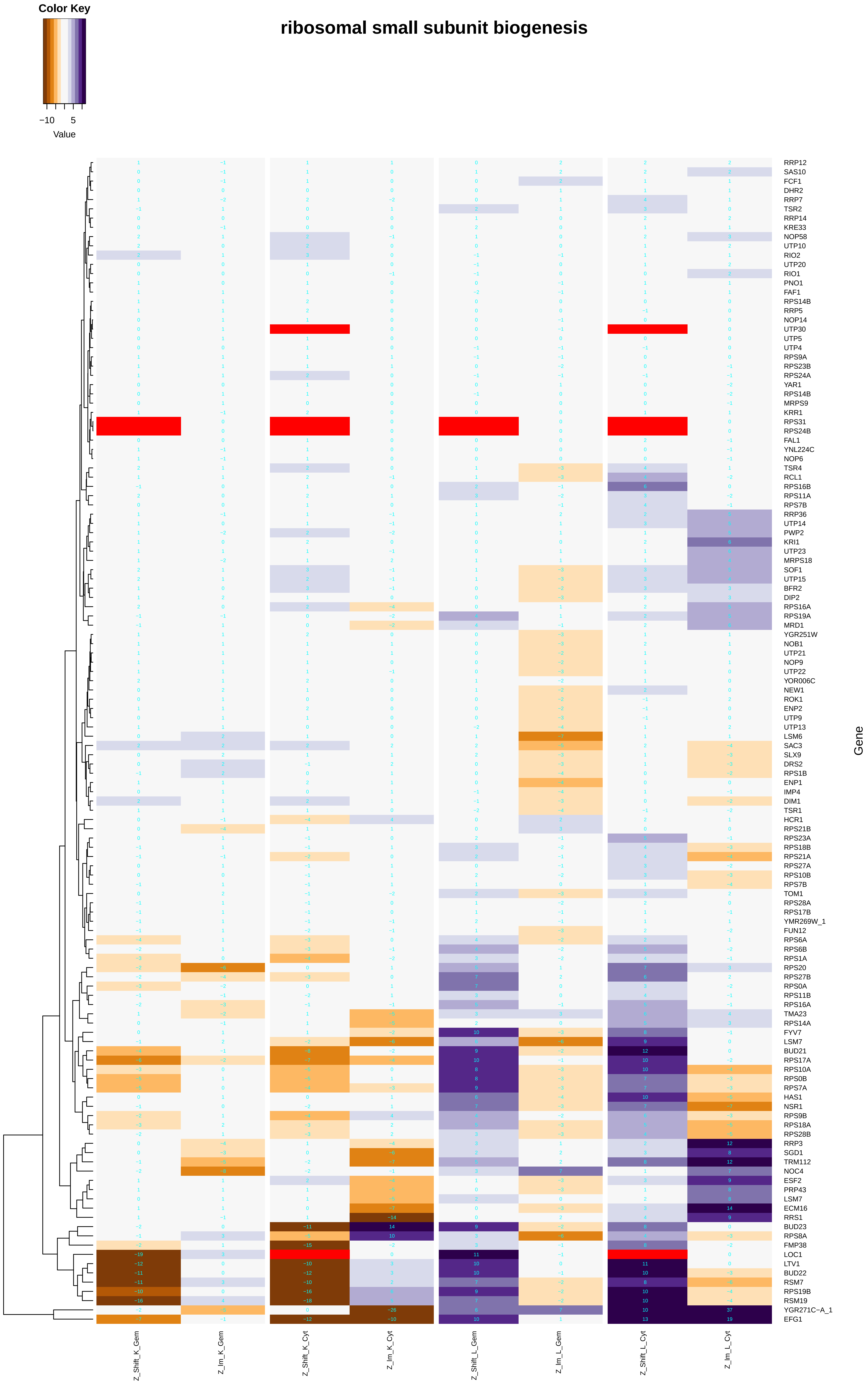
ribosomal small subunit biogenesis
Color Key
−10
5
Value
Gene
RRP12
SAS10
FCF1
DHR2
RRP7
TSR2
RRP14
KRE33
NOP58
UTP10
RIO2
UTP20
RIO1
PNO1
FAF1
RPS14B
RRP5
NOP14
UTP30
UTP5
UTP4
RPS9A
RPS23B
RPS24A
YAR1
RPS14B
MRPS9
KRR1
RPS31
RPS24B
FAL1
YNL224C
NOP6
TSR4
RCL1
RPS16B
RPS11A
RPS7B
RRP36
UTP14
PWP2
KRI1
UTP23
MRPS18
SOF1
UTP15
BFR2
DIP2
RPS16A
RPS19A
MRD1
YGR251W
NOB1
UTP21
NOP9
UTP22
YOR006C
NEW1
ROK1
ENP2
UTP9
UTP13
LSM6
SAC3
SLX9
DRS2
RPS1B
ENP1
IMP4
DIM1
TSR1
HCR1
RPS21B
RPS23A
RPS18B
RPS21A
RPS27A
RPS10B
RPS7B
TOM1
RPS28A
RPS17B
YMR269W_1
FUN12
RPS6A
RPS6B
RPS1A
RPS20
RPS27B
RPS0A
RPS11B
RPS16A
TMA23
RPS14A
FYV7
LSM7
BUD21
RPS17A
RPS10A
RPS0B
RPS7A
HAS1
NSR1
RPS9B
RPS18A
RPS28B
RRP3
SGD1
TRM112
NOC4
ESF2
PRP43
LSM7
ECM16
RRS1
BUD23
RPS8A
FMP38
LOC1
LTV1
BUD22
RSM7
RPS19B
RSM19
YGR271C−A_1
EFG1
Z_Shift_K_Gem
Z_Im_K_Gem
Z_Shift_K_Cyt
Z_Im_K_Cyt
Z_Shift_L_Gem
Z_Im_L_Gem
Z_Shift_L_Cyt
Z_Im_L_Cyt
